# A novel method of sample homogenization with the use of a microtome-cryostat apparatus

**DOI:** 10.1039/c9ra06808b

**Published:** 2019-11-20

**Authors:** Ekaterina A. Zelentsova, Vadim V. Yanshole, Yuri P. Tsentalovich

**Affiliations:** International Tomography Center SB RAS Institutskaya 3a Novosibirsk 630090 Russia yura@tomo.nsc.ru +7-383-333-13-99 +7-383-330-31-36; Novosibirsk State University Pirogova 2 Novosibirsk 630090 Russia

## Abstract

Quantitative metabolomics places high demands on sample preparation, including a high degree of metabolite extraction and controlled sample weight. In respect to elastic collagen-rich tissues, the existing methods of sample homogenization poorly fit these demands due to incomplete homogenization, sample material loss, or metabolite degradation. Herein, a novel method based on the use of a microtome-cryostat apparatus is proposed. The performance of the cryotome method is compared with the results obtained with the use of a vortex bead beating. NMR-based metabolomic analysis shows that the extraction efficiency and the data scattering for both methods of sample preparation are similar. However, the heat generation during the bead beating causes the destruction of thermally-unstable compounds; besides, it may cause protein hydrolysis, leading to an artificial increase in the amino acid level. The cryotome method of sample homogenization does not cause sample heating, and it seems to be ideal for elastic tissues.

## Abstract 2

A novel method of homogenization of elastic tissues does not cause sample heating and material losses.

## Graphical abstract

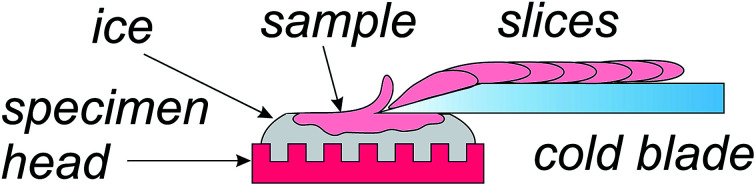

## Introduction

Quantitative metabolomics is probably the most promising and sought-after branch of metabolomics, which is actively being developed and integrated into novel applications in biology and medicine.^1–6^ Unlike qualitative (where only the presence of certain metabolites is detected) and semi-quantitative (the ratio of metabolite levels in the “case” and “control” samples is estimated) metabolomics, quantitative measurements provide the determination of absolute values of metabolite concentrations in a biological tissue (typically, in moles per gram of tissue). The major platforms for metabolomic studies are NMR spectroscopy and liquid or gas chromatography with MS detection.^7,8^ Quantitative metabolomics places high demands on sample preparation, including: a high degree of metabolite extraction from a tissue; sample purification of proteins and lipids to obtain NMR spectra of good quality; and careful sample treatment during the preparation to avoid losses of the sample material or metabolites present in the sample. Unlike the genome or proteome, the metabolome does not contain internal calibrants (such as household proteins) which can be used for the post-correction of the sample size (weight, volume) for the normalization or as a denominator in the concentration calculations. Therefore, the correct determination and preserving of the initial sample size is of vital importance for quantitative metabolomics. The extraction of metabolites from a tissue with controlled sample size is often the most labor-intensive and error-prone step in metabolomic studies. In particular, the correct choice of the solvent mixture for metabolite extraction is extremely important for efficient extraction with the minimal metabolite losses.^9–11^

Depending on the sample type, different methods of the sample preparation are used. Fluids with the low protein content (urine, aqueous humor, tear) can be used with the minimal treatment.^12–17^ Protein-rich and lipid-rich fluids (blood plasma, lymph, milk) require protein precipitation with the use of organic solvents.^18–22^ For NMR-based metabolomics, lipid removal is often desirable, which can be performed by liquid–liquid extraction method such as water/alcohol/chloroform mixtures.^23–25^ The preparation of tissues requires sample homogenization followed by metabolite extraction. The most common method of the sample disruption is the use of mechanical homogenizers,^9,23,24,26^ including glass homogenizers, blenders, rotor-stators, French presses. These methods are optimal for soft tissues such as brain, liver or lens.^18,27,28^ However, it is more difficult to homogenize collagen-rich elastic tissues: blood vessels, ducts, skin. The collagen-rich tissues are usually disrupted by either grinding tissues in liquid nitrogen with mortar and pestle, or by using ball mills or vortexer bead beating. The most important disadvantages of the mortar-and-pestle method are losses of the sample material during the grinding (pieces of sample may fly from the mortar or adhere to the mortar/pestle surface), and changes of the sample weight due to the water condensing on the cold sample, mortar, and pestle. Dust from mortar/pestle produced during the grinding may also change the sample weight. That makes the correct determination of the sample weight difficult, which may induce large errors in the quantitative metabolomics measurements. The limitations of the mortar-and-pestle method also include low throughput and possible sample contamination by the dust produced during the grinding. Ball mills and bead beaters can be considered as the most suitable methods for disruption of elastic and hard tissues.^29,30^ The disadvantage of using ball mills and bead beaters is a possible sample heating, which may result in the decomposition of thermally unstable metabolites. The sample adhesion to the lysing balls or matrix may also introduce an error in the sample weight determination.

In this work, we present a new method of the sample disruption based on the use of a microtome-cryostat apparatus. The general workflow of the method is the following: firstly, we cut the frozen sample into thin slices using the microtome-cryostat. The obtained slices were homogenized in cold methanol with the use of a rotor homogenizer, and the metabolite extraction was performed by water/methanol/chloroform mixture. The method is suitable for preparation of collagen-rich elastic tissues, and its application does not lead to the sample heating or sample material losses. The performance of the method is compared with the sample disruption with the use of a vortexer bead beating. The metabolomic composition of the obtained extracts was measured with the use of ^1^H NMR, which is the most reliable and accurate method for quantification of a moderate number of metabolites in solution.

## Materials and methods

### Materials

All chemicals were purchased from Sigma-Aldrich (USA). Methanol and chloroform HPLC grade were purchased from Scharlau (Spain). H_2_O was deionized using Ultra Clear UV plus TM water system (SG water, Germany) to the quality of 18.2 MΩ. D_2_O 99.9% and sodium 2,2-dimethyl-2-silapentane-5-sulfonate (DSS) were purchased from Cambridge Isotope Laboratories Inc. (USA).

### Sample collection

All procedures of this study were subjected to the Declaration of Helsinki – ethical principles for medical research involving human subjects, and the European Union Directive 2010/63/EU on the protection of animals used for scientific purposes, and with the ethical approval from International Tomography Center SB RAS. Written informed consent was obtained from the patients after explanation of the nature and possible consequences of the study.

Samples of the human breast tissue were obtained from the Novosibirsk City Hospital as the post-operational material after the breast cancer surgery. Only samples with the high content of the ducts (lactiferous ducts under areola area) were used in this study. A sample of the blood vessel (coronary artery) from a farm-bred silver fox (*Vulpes vulpes*) was obtained from the vivarium of the Institute of Cytology and Genetics SB RAS. The samples were frozen immediately after removal, transported to the laboratory and stored at −70 °C until analyzed.

### Sample preparation

Samples were thawed to approximately 0 °C. The breast samples were mechanically cleaned from the fatty tissue with a blunt knife, leaving only the duct tissue. The samples of the duct tissue and the samples of the artery tissue were cut in pieces suitable for further preparation (60–90 mg for duct tissue and 30–40 mg for artery tissue) and weighted. The breast samples from three patients were used for the sample preparation in this study, and all experiments with the artery tissue were performed with the use of a coronary artery from a single animal.

### Homogenization with the use of a cryotome

**Fig. 1:**
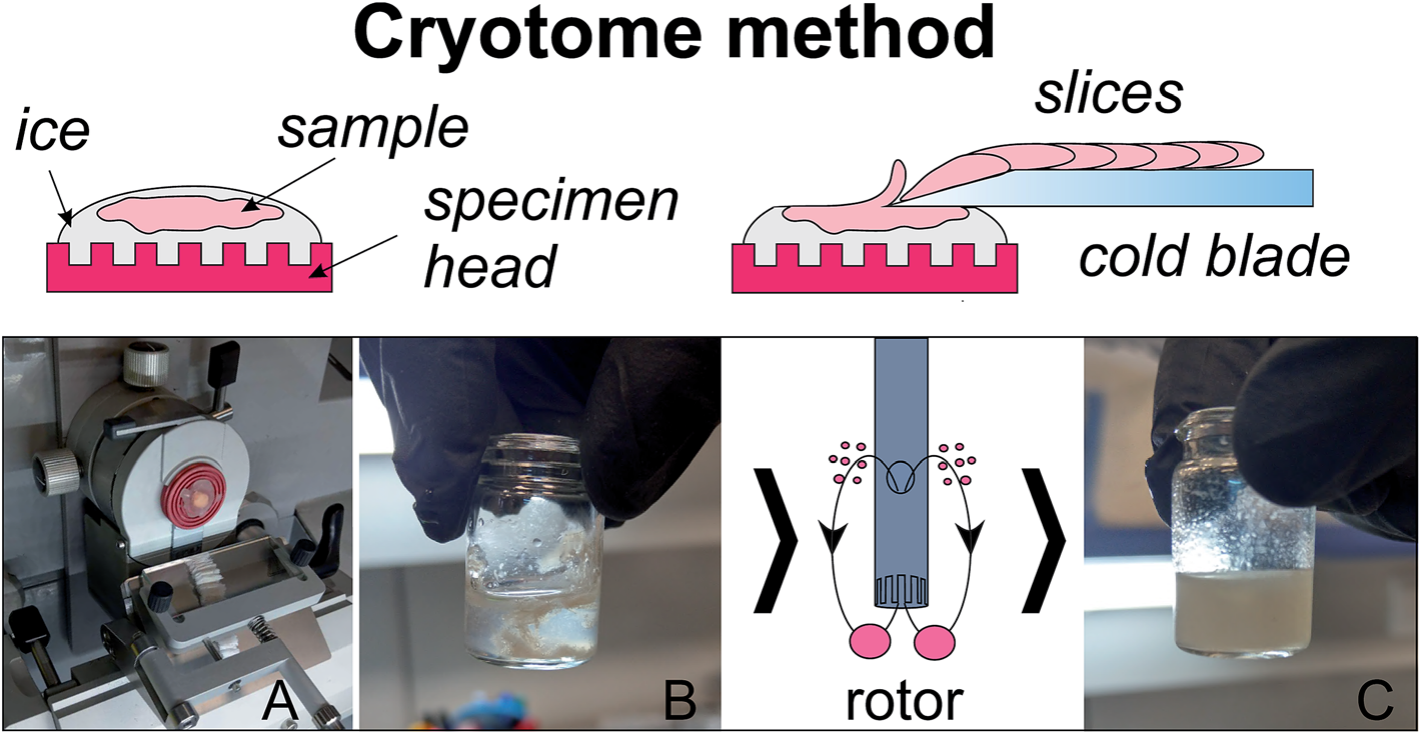
Homogenization with the use of a microtome-cryostat: (A) slicing of a mounted on ice tissue; (B) collecting slices into a vial with addition of cold MeOH; (C) the sample after 10 s of homogenization with rotor homogenizer.

For slicing biological tissues, samples are usually fixed on a specimen head of a cryotome with the conventional embedding media (*e.g.* Neg-50 from Thermo Scientific), consisting mostly of polymers. Pieces of polymer mix with the sample and introduce large chemical noise in the further analysis. For that reason, in the present work the samples were embedded on a specimen head with the use of deionized water as described earlier.^31^ The homogenization of tissues was performed by the following procedure (Fig. 1).

An ice disc of approximately 10 mm diameter and 2–3 mm thickness was formed on the specimen head of a CryoStar NX70 microtome-cryostat (Thermo Scientific, USA) cooled to −16 °C (Fig. 1). A piece of a cold sample (2–5 °C) was placed on a top of the disc and slightly pressed down for several seconds to provide a reliable contact between the disc and the sample. The sample was gently coated with supercooled water. The resulting ice block with the sample frozen inside (Fig. 1A) was sectioned into slices of 5 μm thickness (blade temperature −25 °C). The obtained slices were collected into a glass vial, 1.6 mL of cold methanol (−20 °C) was added (Fig. 1B), and then the sample was homogenized for 10 s (Fig. 1C) with the use of a rotor homogenizer TissueRuptor II (Qiagen, The Netherlands). 0.85 mL of H_2_O and 1.6 mL of cold (−20 °C) chloroform was added to the homogenate to obtain approximately 1 : 2 : 2H_2_O/methanol/chloroform mixture. The mixture was shaken in a shaker for 20 min and left at −20 °C for 20 min. Then the mixture was centrifuged at 16 100*g*, +4 °C for 30 min, the upper (MeOH–H_2_O) layer was collected, lyophilized, and re-dissolved in 0.6 mL of 20 mM deuterated phosphate buffer (pH 7.2) containing 6 × 10^−6^ M of DSS for NMR analysis. The procedure was repeated for a total of seven breast duct samples and seven artery samples.

### Homogenization with the use of a vortexer bead beating

After preliminary estimation of the performance of different types of lysing matrix for the vortexer bead beating recommended by the manufacturer, the matrix A (MP Biomedicals, China) with an additional 1/4 inch zirconium sphere (2 spheres in total) was chosen. A sample together with the matrix and 1 mL of methanol was placed in a sample tube. The sample homogenization was performed with use of a SuperFastPrep-2 bead beater (MP Biomedicals, China). The manufacturer recommends 20 second cycles of vortexing with the sample tube cooling between cycles. To minimize the sample heating, we performed four 5 second cycles with the sample tube cooling on ice between cycles. Then the sample was shaken in a shaker for 20 min, centrifuged at 3000 rpm for 2 min, and the liquid fraction was collected. 0.6 mL of methanol was added to the pellet, shaken for 20 min, centrifuged at 3000 rpm for 2 min, and the liquid fraction was collected again. Then 0.8 mL of H_2_O was added to the remaining pellet, shaken for 20 min, centrifuged at 3000 rpm for 2 min, the liquid fraction was collected, and all three liquid fractions were pooled. 1.6 mL of chloroform was added to the liquid extract to obtain approximately 1 : 2 : 2H_2_O/methanol/chloroform mixture. The future steps of the sample preparation were performed in the same way as for homogenization with the use of a microtome-cryostat. The procedure was repeated for a total of seven duct samples and seven artery samples.

### NMR measurements

The ^1^H NMR measurements were carried out with the use of a NMR spectrometer AVANCE III HD 700 MHz (Bruker BioSpin, Germany) equipped with a 16.44 Tesla Ascend cryomagnet at the Center of Collective Use “Mass spectrometric investigations” SB RAS. The proton NMR spectra for each sample were obtained with 96 accumulations. Temperature of the sample during the data acquisition was kept at 25 °C; the detection pulse was 90 degree. The repetition time between scans was 20 seconds to allow for the relaxation of all spins. Low power radiation at the water resonance frequency was applied prior to acquisition to presaturate the water signal. The concentrations of metabolites in the samples were determined by the peak area integration respectively to the internal standard DSS.

## Results and discussion

**Fig. 2:**
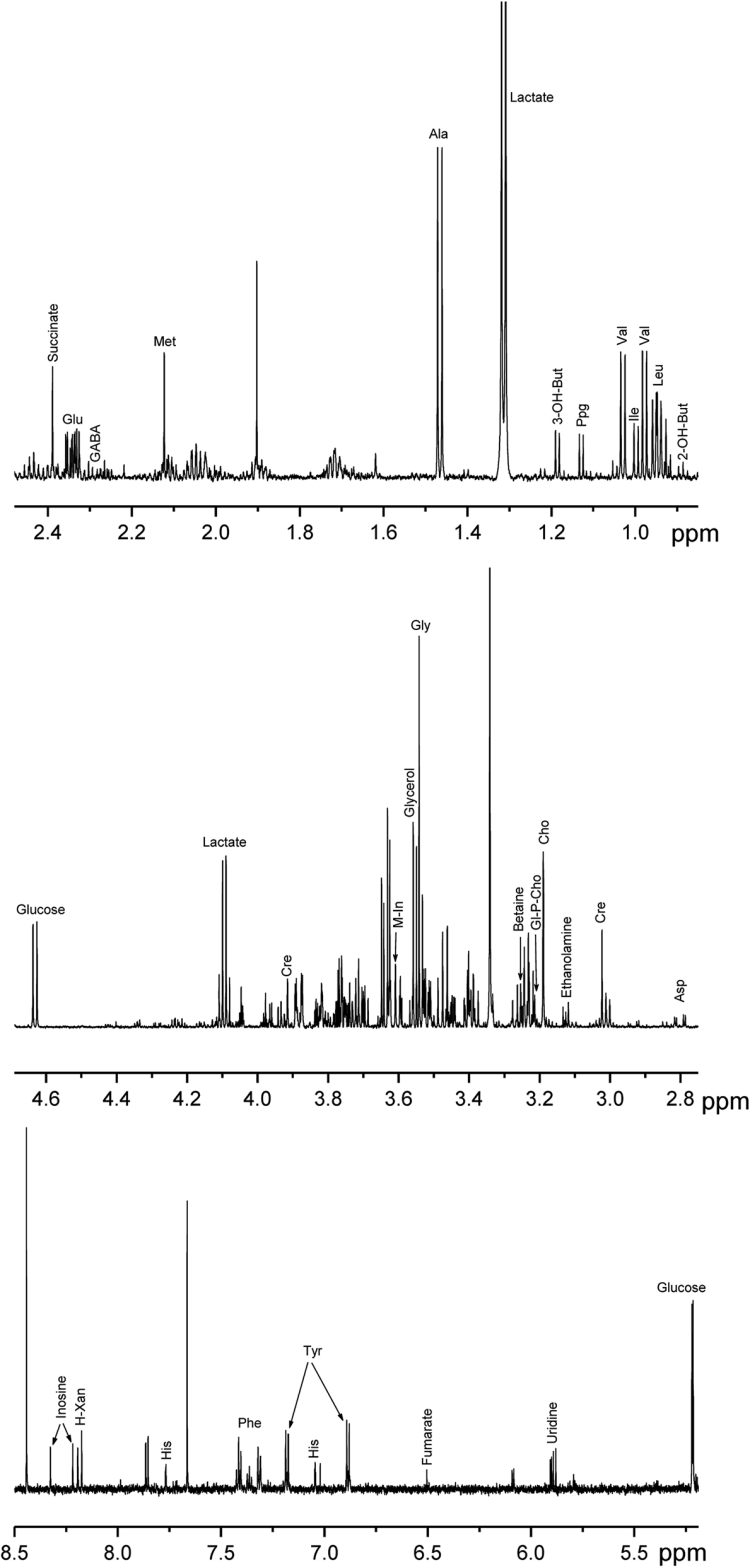
NMR spectrum of a breast duct sample obtained with the use of cryotome method with the assignment of signals taken for the analysis: 2-OH-But – 2-hydroxy-butyrate; 3-OH-But – 3-hydroxy-butyrate; Cho – choline; Cre – creatine; GABA – γ-aminobutyrate; Gl-P-Cho – glycerophosphocholine; H-Xan – hypoxanthine; M-In – *myo*-inositol; Ppg – propylene glycol;. For amino acids, standard 3-letter symbols are used.

**Fig. 3:**
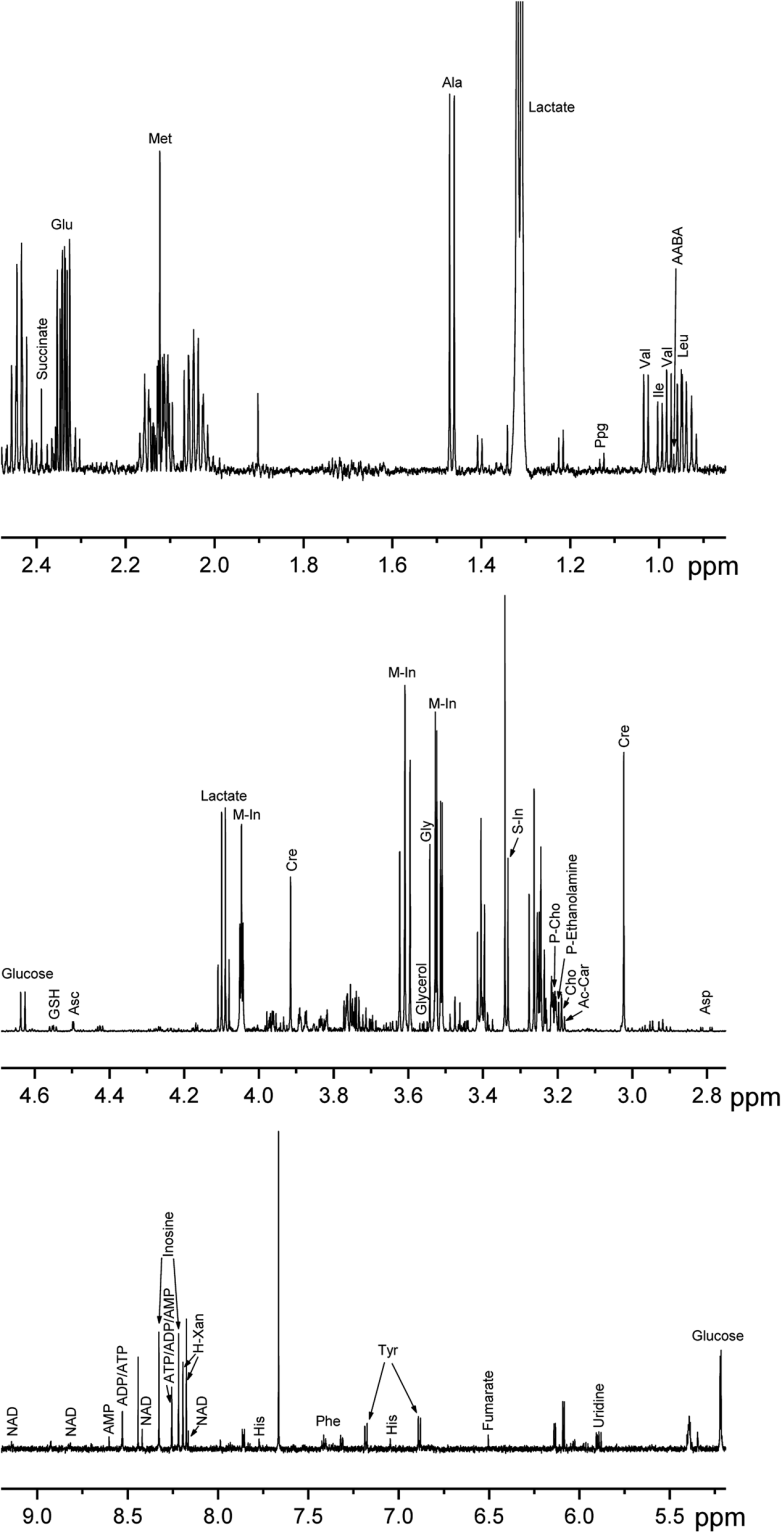
NMR spectrum of an artery sample obtained with the use of cryotome method with the assignment of signals taken for the analysis: AABA – α-aminobutyrate; Ac-Car – acetyl-carnitine; Asc – ascorbate; Cho – choline; Cre – creatine; H-Xan – hypoxanthine; M-In – *myo*-inositol; P-Cho – phosphocholine; P-Ethanolamine – phosphoethanolamine; Ppg – propylene glycol; S-In – *scyllo*-inositol. For amino acids and nucleotides, standard 3-letter symbols are used.

The tissues used in the present work are characterized by high elasticity due to the high collagen content. At the preliminary stage of the study, we tried to homogenize the samples with the use of a glass homogenizer and a rotor homogenizer. The attempts were unsuccessful: even after long homogenization the sample disruption was incomplete with the sample fibers remaining in the homogenate. The use of a vortexer bead beating gave much better results, and we decided to compare the performance of a new method of the sample disruption based on the use of a microtome-cryostat with the performance of the bead beater method. For that purpose, during the sample preparation all samples were divided into pairs: one piece of the breast sample was homogenized using the cryotome method (BC1–BC7 samples), and the adjacent peace of the same breast sample was homogenized by the bead beater method (BB1–BB7 samples). The artery samples were divided in the same way: the neighboring pieces of the same artery were assigned to either microtome-cryostat (AC1–AC7 samples) or bead beater (AB1–AB7) methods. After the sample preparation, the NMR spectra of protein-free lipid-free extracts were obtained, and the concentrations of metabolites in each sample were measured (Fig. 2 and 3).

A typical NMR spectrum of a biological extract contains signals from up to hundred of compounds.^11,16,22,28,32^ However, some of the signals are weak or partly overlapped by other signals; that can cause significant experimental errors in the integration of these signals. For that reason, only 28 (breast tissue) and 34 (artery tissue) metabolites giving strong and distinct signals in the NMR spectra were chosen for the comparative analysis of performance of different homogenization methods. The signal assignment of these compounds in NMR spectra was performed in our previous publications^15–17,28^ using combined NMR and LC-MS measurements and spiking the extracts with chemical standards (“Level 1” of identification according to Metabolomics Standards Initiative guidelines); it is shown in Fig. 2 and 3.

Tables 1 and 2 contain the data of the comparative metabolomic analysis of the breast (Table 1) and artery (Table 2) samples homogenized by two methods. In Tables, the second and the third columns show the average values and standard deviations (SD) of metabolite concentrations in the samples homogenized by the cryotome and bead beater methods. The ratios of the metabolite concentrations in the neighboring pieces from the same tissue sample prepared by two methods (BC1/BB1–BC7/BB7; AC1/AB1–AC7/AB7) were calculated, and the average values (BC/BB)_mean_ and (AC/AB)_mean_ and their SDs are presented in the fourth column. The relative standard deviation (RSD = SD/mean), characterizes the data scattering typical for the method used; the RSD values are given in the fifth and sixth columns. In the calculation of the average values for AC and AB samples, last five metabolites demonstrating thermal decomposition (see below) were excluded from calculation.

**Table 1:** Concentrations of metabolites in breast samples determined with the use of cryotome (BC samples) and bead beater (BB samples) methods^a^

Metabolite	BC samples, nmol g^−1^	BB samples, nmol g^−1^	Mean ratio BC/BB ± SD	RSD for BC samples	RSD for BB samples
Inosine	71 ± 27	70 ± 20	0.9 ± 0.4	0.37	0.29
Phenylalanine	120 ± 50	120 ± 30	1.0 ± 0.2	0.44	0.27
Alanine	570 ± 170	590 ± 140	1.0 ± 0.1	0.31	0.25
Leucine	190 ± 70	180 ± 40	1.0 ± 0.2	0.35	0.23
Valine	240 ± 60	230 ± 50	1.0 ± 0.2	0.24	0.19
Succinate	78 ± 25	74 ± 11	1.0 ± 0.2	0.32	0.14
Glycerophosphocholine	32 ± 15	31 ± 14	1.0 ± 0.3	0.48	0.46
Histidine	96 ± 21	93 ± 11	1.0 ± 0.2	0.22	0.12
Glycerol	800 ± 700	780 ± 700	1.0 ± 0.1	0.87	0.89
2-Hydroxy-butyrate	49 ± 11	49 ± 8	1.0 ± 0.3	0.22	0.16
3-Hydroxy-butyrate	260 ± 200	240 ± 160	1.0 ± 0.1	0.77	0.70
Glucose	4000 ± 900	3700 ± 600	1.1 ± 0.1	0.22	0.16
GABA	47 ± 28	44 ± 27	1.1 ± 0.3	0.59	0.61
Betaine	36 ± 15	34 ± 14	1.1 ± 0.1	0.43	0.41
Uridine	100 ± 20	98 ± 24	1.1 ± 0.3	0.20	0.24
Glycine	790 ± 320	740 ± 320	1.1 ± 0.2	0.41	0.43
Isoleucine	96 ± 35	86 ± 23	1.1 ± 0.2	0.36	0.27
Propylene glycol	140 ± 40	130 ± 30	1.1 ± 0.2	0.26	0.20
Methionine	76 ± 30	68 ± 18	1.1 ± 0.3	0.39	0.26
Tyrosine	130 ± 40	120 ± 30	1.1 ± 0.3	0.31	0.28
Hypoxanthine	73 ± 41	62 ± 25	1.1 ± 0.3	0.56	0.41
Ethanolamine	200 ± 60	170 ± 30	1.2 ± 0.3	0.30	0.18
*myo*-Inositol	520 ± 170	450 ± 140	1.2 ± 0.2*	0.33	0.30
Creatine	210 ± 60	180 ± 50	1.2 ± 0.1*	0.29	0.29
Lactate	3000 ± 1000	2400 ± 900	1.2 ± 0.1*	0.35	0.36
Glutamate	530 ± 110	440 ± 110	1.2 ± 0.3	0.21	0.25
Choline	280 ± 110	220 ± 90	1.3 ± 0.4*	0.37	0.41
Aspartate	98 ± 66	61 ± 37	1.4 ± 0.3*	0.68	0.61
Average value			1.10	0.39	0.33

a * Indicates statistically significant difference according to the Student's test.

**Table 2:** Concentrations of metabolites in artery samples determined with the use of cryotome (AC samples) and bead beater (AB samples) methods^a^

Metabolite	AC samples, nmol g^−1^	AB samples, nmol g^−1^	Mean ratio AC/AB ± SD	RSD for AC samples	RSD for AB samples
Fumarate	15 ± 4	29 ± 5	0.5 ± 0.1*	0.24	0.18
Valine	150 ± 10	250 ± 20	0.6 ± 0.1*	0.09	0.10
Leucine	190 ± 20	300 ± 30	0.6 ± 0.1*	0.10	0.09
Tyrosine	140 ± 20	210 ± 20	0.7 ± 0.1*	0.16	0.11
Isoleucine	110 ± 10	170 ± 30	0.7 ± 0.1*	0.10	0.17
Hypoxanthine	430 ± 50	620 ± 100	0.7 ± 0.1*	0.12	0.16
Nicotinamide	50 ± 15	78 ± 21	0.7 ± 0.1*	0.30	0.27
Phenylalanine	90 ± 18	140 ± 30	0.7 ± 0.2*	0.20	0.19
Glycerol	210 ± 30	320 ± 80	0.7 ± 0.1*	0.12	0.27
Inosine	320 ± 30	460 ± 40	0.7 ± 0.1*	0.09	0.08
Alanine	600 ± 50	850 ± 60	0.7 ± 0.1*	0.08	0.07
Histidine	83 ± 12	120 ± 20	0.7 ± 0.1*	0.14	0.15
Methionine	210 ± 20	280 ± 30	0.8 ± 0.1*	0.08	0.10
Glucose	2800 ± 600	3100 ± 800	0.9 ± 0.1	0.21	0.24
Aspartate	110 ± 20	130 ± 20	0.9 ± 0.3	0.17	0.15
Glycine	1200 ± 100	1300 ± 100	0.9 ± 0.1	0.11	0.09
Acetyl-carnitine	31 ± 2	33 ± 4	0.9 ± 0.1	0.07	0.11
Glutamate	1700 ± 200	1900 ± 300	0.9 ± 0.1	0.10	0.17
Creatine	1600 ± 100	1600 ± 100	1.0 ± 0.1	0.08	0.09
Lactate	9500 ± 1700	9900 ± 2800	1.0 ± 0.2	0.18	0.29
*Myo*-Inositol	8100 ± 1000	8100 ± 1300	1.0 ± 0.1	0.12	0.17
*Scyllo*-Inositol	350 ± 50	350 ± 70	1.0 ± 0.1	0.15	0.19
Choline	110 ± 10	110 ± 20	1.1 ± 0.3	0.11	0.19
Ascorbate	100 ± 56	83 ± 34	1.1 ± 0.3	0.56	0.41
Phosphocholine	110 ± 20	85 ± 13	1.3 ± 0.4	0.21	0.15
Uridine	100 ± 16	79 ± 11	1.3 ± 0.4	0.16	0.14
Phosphoethanolamine	1000 ± 100	730 ± 140	1.4 ± 0.2*	0.12	0.19
Propylene glycol	39 ± 12	25 ± 12	1.9 ± 0.9*	0.32	0.49
Succinate	39 ± 7	23 ± 10	1.9 ± 0.7*	0.18	0.42
ATP	42 ± 8	2 ± 2	8 ± 3*	0.19	0.31
NAD	54 ± 20	6 ± 3	9 ± 4*	0.37	0.43
GSH	330 ± 120	20 ± 10	15 ± 4*	0.37	0.54
AMP	37 ± 15	2 ± 1	22 ± 6*	0.40	0.44
ADP	200 ± 40	6 ± 4	62 ± 35*	0.18	0.63
Average value			0.95^b^	0.16^b^	0.19^b^

a * Indicates statistically significant difference according to the Student's test.

b In the calculation of the average value, last five metabolites were excluded.

The comparison of the data for breast samples given in Table 1 demonstrates similar performances of both methods of the sample homogenization. On average, the extraction efficiency of the microtome-cryostat method is better by approximately 10%, but the statistically significant difference (according to the Student's test) was found only for few metabolites (marked by * in Tables 1 and 2). On the other hand, the data variation characterized by the RSD value for the bead beating method is slightly lower than that for cryotome method (on average, 0.33 *versus* 0.39). We should notice that the breast samples were taken from three donors, and the observed data variation may correspond to the intersample difference rather than to the experimental error.

**Fig. 4:**
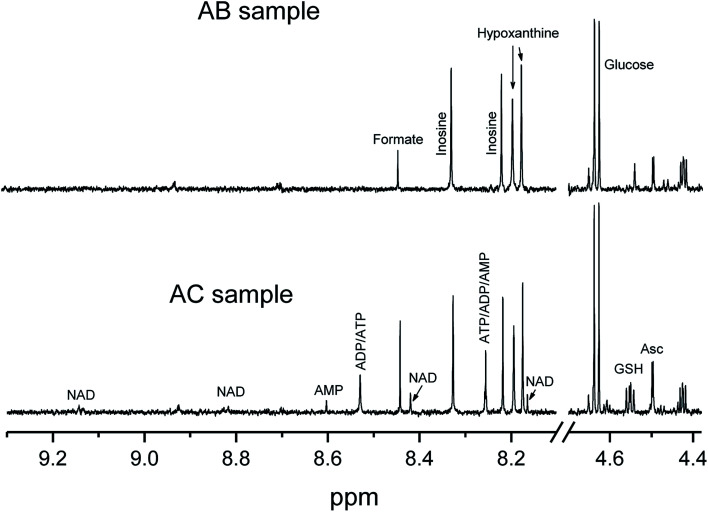
Selected regions of NMR spectra of artery extracts obtained with the use of bead beater (AB sample) and cryotome (AC sample) methods with the signal assignment: Asc – ascorbate. For amino acids and nucleotides, standard 3-letter symbols are used.

The difference in the method performances for the artery samples is significantly more pronounced (Table 2). Firstly, for a group of compounds their levels in AC samples are much higher than that in AB samples. The data on these metabolites are absent in Table 1 because their concentrations in all breast samples were below measurable level. This group includes nucleotides ATP, ADP, AMP, and NAD, and antioxidant GSH. In fact, for some AB samples the signals from these compounds were hardly visible in the NMR spectra (Fig. 4). Most likely, these metabolites underwent thermal decomposition during the bead beating, although we used very gentle conditions: the beating cycle was only 5 seconds instead of 20 seconds recommended by manufacturer. The mechanism of the metabolite decomposition is not obvious: the temperature of the sample tube immediately after the vortexing did not exceed 35–40 °C, which is not sufficient for the fast thermal degradation of metabolites. Very likely that the high temperature spots are created at the points of the friction of matrix particles and sphere hits, and the metabolite decomposition takes place in these spots before the heat dissipated in the sample volume. It is also possible that chemically or biologically active components in the tissue homogenate (enzymes, oxidizing agents including molecular oxygen, metal ions) participate in the reactions of metabolite decomposition. Secondly, there is a group of compounds which concentrations in the samples prepared with the bead beating method (AB samples) is 1.3–2 times higher than in the samples prepared with the cryostat method (AC samples). This group mostly consists of proteogenic amino acids. These observations might be attributed to the better sample homogenization with the bead beating method, and, correspondingly, to the better metabolite extraction; however, it is more likely that the sample heating during the bead beating causes the protein hydrolysis^33,34^ leading to the artificial increase of amino acid concentrations. Interestingly that in the preparation of the breast duct tissue with cryotome and beat beating methods, no difference for these compounds has been observed. Probably, that means that proteins in the duct tissue are more stable than proteins in the artery tissue, and do not undergo hydrolysis. Another possible explanation is that the vessel tissue is more viscous than the duct tissue, and the heat generation is higher.

The concern about degradation of unstable metabolites during the sample preparation has been expressed in several recent publications.^35,36^ To validate the hypothesis of the thermal metabolite degradation and protein hydrolysis, we performed control experiments. Firstly, the artery samples were disrupted using the cryotome method, the obtained homogenates were incubated at 60 °C for 20 and 40 minutes. Then the metabolite extraction was performed, and the NMR spectra of extracts were obtained. It was found the concentrations of ATP, ADP, AMP, NAD, and GSH under incubation significantly drop, while the concentrations of proteogenic amino acids increase. In the second experimental run, the extraction and the incubation were reversed in time: already extracted metabolites in methanol-water solution were subjected to incubation. In this case, the concentrations of nucleotides and amino acids remained almost unchanged. These results confirm that indeed, the increase of the concentrations of proteogenic amino acids after bead beating should be attributed to the thermal protein hydrolysis, and the decrease of nucleotide concentrations – to their thermal degradation accelerated by biological components present in a tissue.

All artery samples were prepared from the same artery specimen, and the data variation is significantly lower than that for breast samples: the average RSD values are 0.16 and 0.19 for cryostat and bead beating methods, respectively. We suppose that these values are a good estimation of the combined experimental errors of the methods, including errors in the sample weighting, sample preparation, and NMR measurements.

The comparison of vortexer bead beating and cryotome methods shows the advantages of the latter. Although the extraction efficiencies of both methods for majority of metabolites are similar, the cryotome method does not cause the sample heating, which may result in the decomposition of unstable metabolites and the artificial increase of amino acid concentrations due to the protein hydrolysis. The cryotome method allows for the exact determining of the sample weight, and the material losses during the sample preparation are minimal; that is very important for quantitative measurements. Taking into account that other methods of the sample disruption (glass homogenizers, rotor homogenizers, grinding with mortar and pestle) are poorly suitable for elastic tissues with the high collagen content, the cryotome method seems to be ideal for such tissues. It is important that the application of the cryotome method does not lead to a significant decrease of throughput of the sample preparation: the laboriousness of the sample preparation with the use of a cryotome and a rotor homogenizer is approximately 20–25% higher than that with the use of a rotor homogenizer only. More precisely, the preparation of ten samples with the use of the rotor homogenizer takes approximately 2.5 hours, and that with the use of the bead beater, or the cryotome and rotor homogenizer – approximately 3 hours. We developed this method for the sample preparation for quantitative metabolomics; however, the method can be also successfully used for other applications, including proteomics, semi-quantitative and qualitative metabolomics. Metabolomic profiling of breast and vessel tissues plays an increasing role in the study of oncologic and cardiovascular diseases,^37–39^ and the method can be a useful tool for medical applications.

## Conflicts of interest

The authors declare no conflict of interest.

## Supplementary Material
